# A novel myeloid cell marker genes related signature can indicate immune infiltration and predict prognosis of hepatocellular carcinoma: Integrated analysis of bulk and single-cell RNA sequencing

**DOI:** 10.3389/fmolb.2023.1118377

**Published:** 2023-03-07

**Authors:** Su-Su Zheng, Yan-Fang Wu, Bo-Heng Zhang, Cheng Huang, Tong-Chun Xue

**Affiliations:** ^1^ Department of Hepatic Oncology, Xiamen Clinical Research Center for Cancer Therapy, Zhongshan Hospital, Fudan University (Xiamen Branch), Xiamen, China; ^2^ The Liver Cancer Institute, Zhongshan Hospital and Shanghai Medical School, Fudan University, Key Laboratory for Carcinogenesis and Cancer Invasion, The Chinese Ministry of Education, Shanghai, China; ^3^ Center for Evidence-based Medicine, Shanghai Medical School, Fudan University, Shanghai, China; ^4^ Department of Liver Surgery, Liver Cancer Institute, Zhongshan Hospital, and Key Laboratory of Carcinogenesis and Cancer Invasion (Ministry of Education), Fudan University, Shanghai, China

**Keywords:** tumor associated myeloid cells, single-cell RNA sequencing, hepatocellular carcinoma, prognosis, immune infiltration, drug resistance

## Abstract

Myeloid cells are physiologically related to innate immunity and inflammation. Tumor-associated myeloid cells gained increasing interest because of their critical roles in tumor progression and anticancer immune responses in human malignancies. However, the associations between tumor-associated myeloid cell-related genes and hepatocellular carcinoma have yet to be revealed. Here, through the integrating analysis of bulk and single-cell RNA (scRNA) sequencing of public HCC samples, we developed a gene signature to investigate the role of HCC-specific myeloid signature genes in HCC patients. We firstly defined 317 myeloid cell marker genes through analyzing scRNA data of HCC from the GEO dataset. After selecting the differentially expressed genes, eleven genes were also proved prognostic. Then we built a gene signature from the TCGA cohort and verified further with the ICGC dataset by applying the LASSO Cox method. An eight genes signature (FABP5, C15orf48, PABPC1, TUBA1B, AKR1C3, NQO1, AKR1B10, SPP1) was achieved finally. Patients in the high risk group correlated with higher tumor stages and poor survival than those in the low-risk group. The risk score was proved to be an independent risk factor for prognosis. The high risk group had higher infiltrations of dendritic cells, macrophages and Tregs. And the APC co-inhibition, T cell co-inhibition pathways were also activated. Besides, the risk score positively correlated with multidrug resistance proteins. In conclusion, our myeloid cell marker genes related signature can predict patients’ survival and may also indicate the levels of immune infiltration and drug resistance.

## Introduction

Hepatocellular carcinoma accounts for the majority of liver cancer and is one of the leading cause for cancer-related deaths globally (Sung et al., 2021). Further, most patients lost the chance of surgery because of vascular invasion or even metastasis. For these advanced HCC, systemic treatments including immune treatment are highly recommended (Sharafi et al., 2022). Among various solid tumors, immune treatment is especially widely used in HCC. Immune checkpoint inhibitors treatment was highly recommended to combine with TKI or bevacizumab. The combination of ICIs and tyrosine kinase inhibitor/bevacizumab therapy has gained synergistic effects (Finn et al., 2020; Roskoski, 2022). However, until now, there are no well-established indicators of immunotherapy response for HCC because of limited knowledge about the immune-related tumor microenvironment.

Myeloid cells are physiologically related to innate immunity and inflammation for cancer. Tumor associated myeloid cells, as an important part of the TME, can be divided into monocytes/macrophages, myeloid-derived suppressor cells, dendritic cells, and granulocytes (Bassler et al., 2019). In recent decades, the exact roles of TAMCs have gained increasing interest since these cells are important indicators for treatment efficacy and disease prognosis in patients. For example, the density of TAMCs are commonly negatively correlated with patients’ survival, while some special subtypes of TAMCs (M1 subtype macrophage, N2 subtype neutrophil) are proved to be associated with better prognosis (DeNardo and Ruffell, 2019; Engblom et al., 2016; Fridlender et al., 2009; Locati et al., 2020; Nakamura and Smyth, 2020). In HCC, our previous work showed that the high post-treatment neutrophil-to-lymphocyte ratio indicated a higher risk of metastasis for patients undergoing transarterial chemoembolization (Xue et al., 2015). Especially, the TME infiltrating neutrophils, as one kind of innate immune cells have been shown to be involved in treatment and prognosis among HCC patients (Chen et al., 2021). Due to the flexibility of TAMCs, it is necessary to develop gene signatures to evaluate their roles in immune infiltration and predict patients’ prognosis. Through high-throughput sequencing technologies, we are now able to perform analysis to comprehensively catalog myeloid cells related genes in cancers. Liu et al. developed a myeloid cells related gene signature containing 5 genes, which was valuable in evaluating the prognosis and immunity for head and neck squamous cell carcinoma (Liu et al., 2021). However, their myeloid signature genes were generated from published literature of various tumors. To the best of our knowledge, the HCC specific myeloid cells marker genes related signature has not yet been reported.

Through single-cell RNA-sequencing (scRNA-seq) method, we may further uncover the molecular characteristics of HCC associated myeloid cells in the TME (Chen et al., 2019). In the present study, we firstly identified the tumor associated myeloid cells related marker genes with scRNA-seq analysis of HCC samples from the Gene Expression Omnibus (GEO) dataset. And then, a myeloid cell marker genes related signature was built and validated as an independent risk factor. Besides, the risk score was proved to be associated with immune infiltration and immune-suppressive microenvironment. Moreover, the relationship between risk scores and drug sensitivity was further evaluated.

## Materials and methods

### Data source

ScRNA-seq data of 7 tumor samples from 2 HCC patients was obtained from the GEO dataset, namely, GSE112271. RNA sequencing data of (fragment per kilobase million, FPKM) was obtained from the TCGA database (https://portal.gdc.cancer.gov/repository), which included 374 HCC patients. The International Cancer Genome Consortium (ICGC) database (https://icgc.org/) contained 231 HCC samples. And the TCGA database was used for model construction, while the ICGC database for model validation. All datasets were available from public websites, and ethics approval was confirmed to be obtained from original studies.

### Achieving HCC-specific myeloid cell marker genes

The scRNA-seq data from GSE112271 has been fully described by Bojan Losic et al. (Losic et al., 2020). Here, we combined data from all 7 samples derived from 2 HCC patients. The data was then analyzed with the R software by using the Seurat package (Butler et al., 2018). Quality control was performed as the following: gene numbers more than 500, mitochondrial gene percentage no more than 20, and total UMI counts more than 1,000 (Guan et al., 2022). The “RunHarmony” function in R package “harmony” was then applied to remove the batch effects (Korsunsky et al., 2019). Principal component analysis (PCA) was conducted with the top 2000 variable genes. And cell clusters were defined with the method of shared nearest neighbor (SNN), and the resolution was set as 0.5. The uniform manifold approximation and projection (UMAP) analysis was introduced for visualization. The differentially expressed genes (DEGs) were identified with the “FindAllMarkers” function. The cutoff threshold was set as an adjusted *p*-value <0.01 and |log2 (fold change)| >1. Cell clusters were annotated by using data from published literature as well as the Human Primary Cell Atlas (Losic et al., 2020; Mabbott et al., 2013; Sun et al., 2021).

### Construction and validation of a myeloid marker genes related gene signature

The cutoff threshold was set as an adjusted *p*-value <0.01 and |log2 (fold change)| >1.5 for identifying DEGs between tumor tissues and non-tumor tissues with the “limma” R package in TCGA dataset. The prognostic value of myeloid marker genes was further analyzed by Univariate Cox. Intersection genes were acquired from DEGs and prognostic genes and were then used for model construction with the method of least absolute shrinkage and selection operator (LASSO) Cox regression (Tibshirani, 1997).Then, an eight myeloid marker genes related gene signature was constructed. For each patient, a risk score was calculated by using his gene expression and the regression coefficient. According to the median risk score, all patients were divided into high and low-risk groups. The “survminer” and “timeROC” R package was adopted for survival analysis and predictive accuracy. The ICGC dataset was used for verification with the same formula. Besides, clinical factors such as age, gender, and tumor stage were manually extracted from both the TCGA and ICGC datasets, and were applied for univariate and multivariable Cox regression analysis.

### Patients and immunohistochemical staining

Seventy tumor tissues were obtained from 70 HCC patients in our liver cancer institute. SPP1, CD11b, CD4, and CD8 were selected for protein validation by immunohistochemistry. The expression intensity of SPP1 was evaluated according to a previous research (Wu et al., 2010). The number of positive cells were counted as previously reported (Deng et al., 2017). The study was approved by the Ethics Committee of Zhongshan Hospital, Fudan University.

### Functional enrichment analysis

Kyoto Encyclopedia of Genes and Genomes (KEGG) analyses were performed by gene set enrichment analysis (GSEA) with GSEA software 4.0.1 according to the high and low-risk group. And the top 50 pathways between different groups were exhibited.

### Immune infiltration, TME and drug sensitivity analysis

Immune infiltration analysis was conducted with the “GSVA” R package. Single-sample gene set enrichment analysis (ssGSEA) was adopted for calculating the infiltration scores of immune cells and immune-related pathways (Newman et al., 2019). The association between risk score and pan-cancer immune infiltrates was also compared (Tamborero et al., 2018). Stromal and immune cell infiltration scores were also analyzed with the “ESTIMATE” R package (Yoshihara et al., 2013). Correlations between multidrug resistance genes and risk scores were also analyzed.

### Statistical analysis

DEGs between different groups were compared by Wilcoxon test. The ssGSEA scores between different groups were compared by Mann-Whitney test, and the adjusted *p*-values were generated by using Benjamini–Hochberg method. Survival analysis was conducted with the Kaplan-Meier method. Univariate and multivariate Cox analyses were utilized for identifying prognostic factors. *p*-value < 0.05 was considered to be statistical significance if not specified. All data analyses and figures were accomplished with the R software (version 4.1.3).

## Results

### Identification of HCC-specific myeloid cell marker genes

**FIGURE 1:**
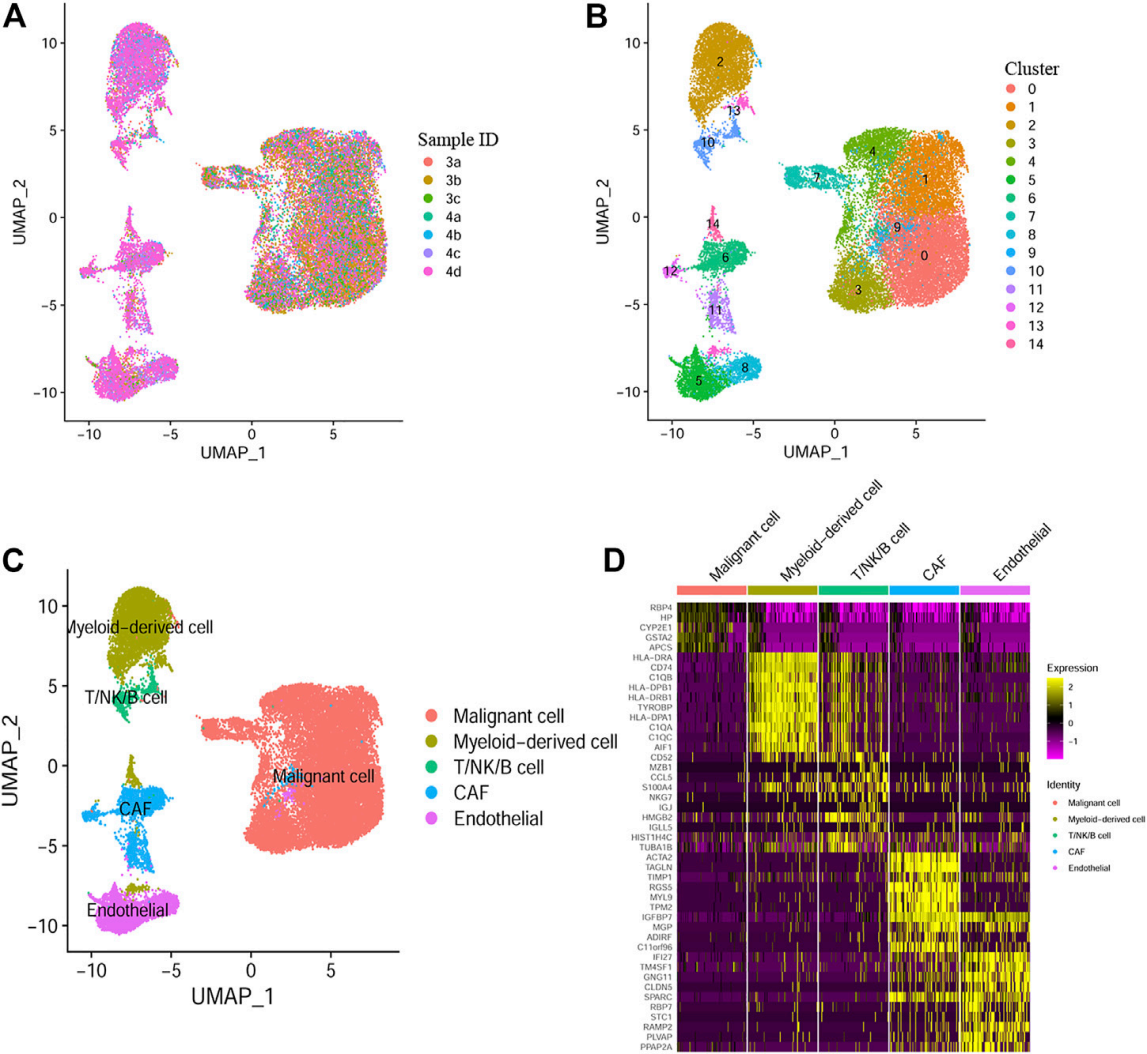
Myeloid cell marker genes defined by Single-cell RNA sequencing **(A)** UMAP plot colored by 7 HCC samples. **(B)** UMAP plot colored by 15 cell clusters. **(C)** Cell types defined by known cell marker genes. **(D)** Heatmap exhibiting the top 10 marker genes for each cell type.

Through scRNA-seq data analysis of GSE112271, we obtained 30054 eligible cells from seven samples of two HCC patients and were further divided into 15 clusters (Figures 1A, B). Next, we annotated the cell clusters with data from published literature and the Human Primary Cell Atlas. Similar to the results of Bojan Losic et al., 7 cell types were discovered, including Malignant cell (ALB, FGG), myeloid-derived cell (HLA-DQB1, CD68), cancer-associated fibroblasts (ACTA2, TAGL), endothelial cell (VWF, KDR), T cell (CD3D, CD2), B cell (CD79A, IGJ) and NK cell (CD69, GNLY). Cells in cluster 2, 13, and 14 were defined as myeloid-derived cells (Figure 1C). By using the “FindAllMarkers” function, marker genes belonging to different cell type were defined, the top 10 genes were shown in Figure 1D. Finally, we identified 317 HCC-specific myeloid cell marker genes (Supplementary Table S1). The flow chart of the study design and analysis was indicated in Supplementary Figure S1.

### Construction and validation of a myeloid cell marker genes related gene signature

**FIGURE 2:**
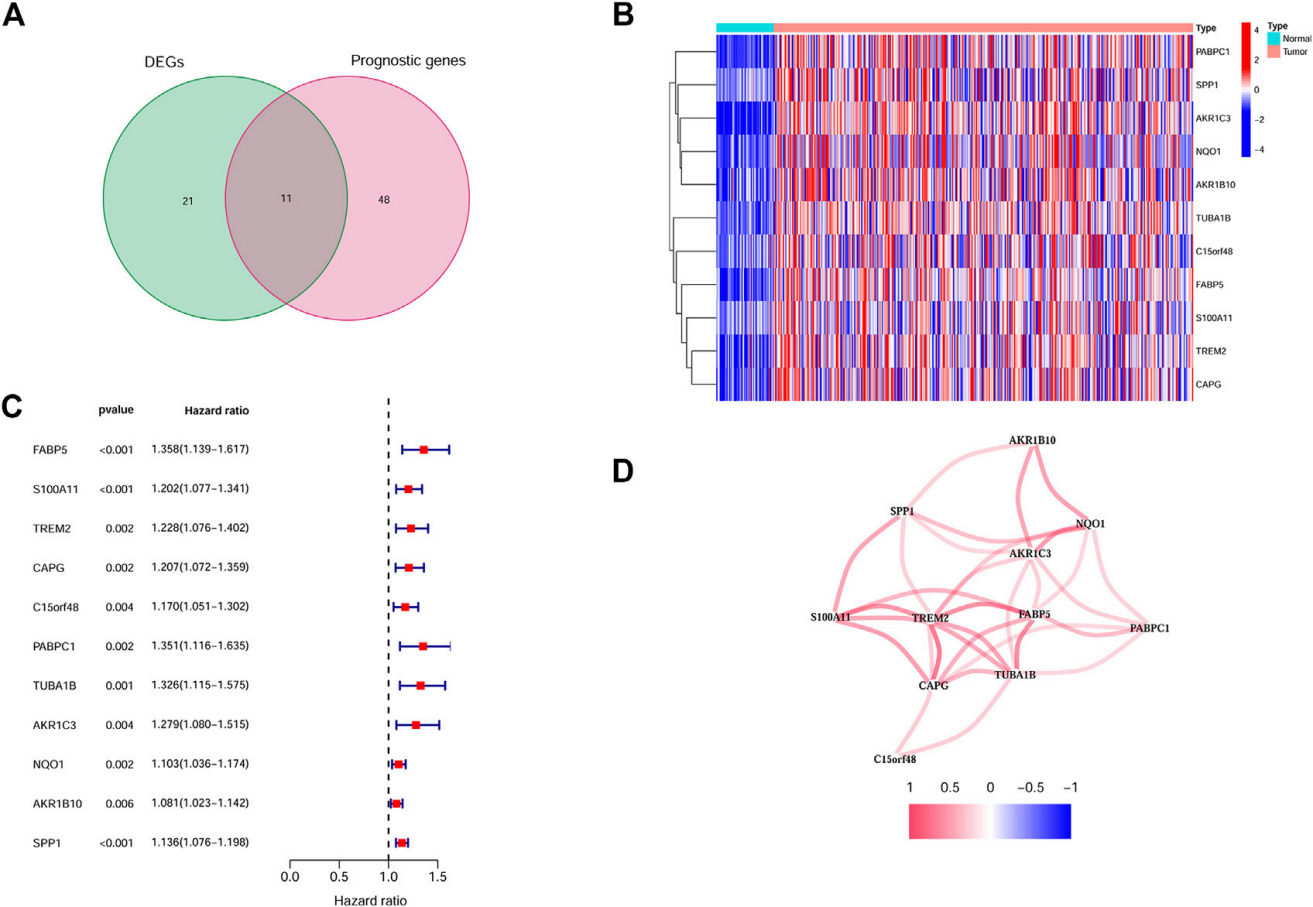
Identification of the candidate Myeloid cell marker genes. **(A)** Venn plot showing the intersection genes. **(B)** Heatmap showing the expression of the 11 intersection genes. **(C)** Forest plots showing the prognostic value of the 11 intersection genes. **(D)** The correlations between 11 selected genes.

Based on the TCGA cohort, we firstly identified 32 DEGs between the tumor and non-tumor tissues. At the same time, by univariate Cox analysis, 11 genes were finally selected to be both differently expressed and prognostic (Figure 2A). The expression of these genes was shown as a heatmap (Figure 2B). The hazard ratio of each gene was shown in Figure 2C. The correlations among different genes were exhibited in Figure 2D. Next, through the LASSO Cox regression analysis, we constructed an 8 genes signature, the calculation function of risk score was set as follows: Riskscore = (0.06*FABP5 expression) + (0.064*C15orf48 expression) + (0.063*PABPC1 expression) + (0.103*TUBA1B expression) + (0.019*AKR1C3 expression) + (0.015*NQO1 expression) + (0.005*AKR1B10 expression) + (0.07*SPP1 expression). We next selected the top 3 genes with highest weights (TUBA1B/SPP1/C15orf48) and validated the correlation between their expression and the infiltration of myeloid cell in HCC with the online tool Timer (https://cistrome.shinyapps.io/timer/). And the results showed that all three genes were positively correlated with the infiltration of macrophage, neutrophil, and dendritic cell (Supplementary Figure S2). Moreover, SPP1 protein level was further investigated by immunohistochemistry, and the association between SPP1 and tumor-infiltrating immune cells was explored. As shown in Supplementary Figure S3, SPP1 was positively correlated with the infiltration of myeloid cells (indicated as CD11b positive cells), and negatively correlated with CD4/CD8 cells.

**FIGURE 3:**
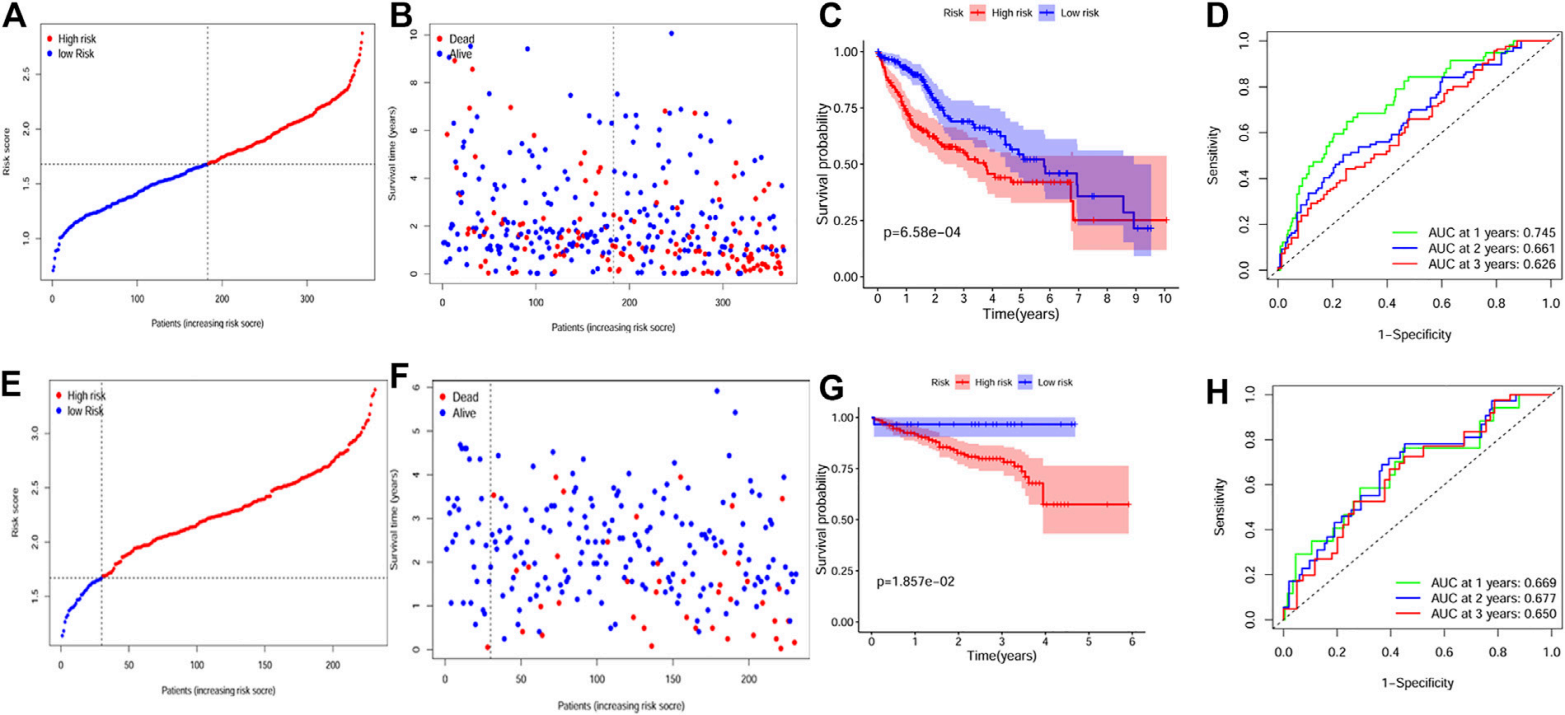
Construction and validation of the gene signature. **(A, B)** The distribution of risk score and survival status for the TCGA dataset. **(C)** Survival analysis between the high and low risk groups for the TCGA dataset. **(D)** ROC curves showing the predictive value of the risk score for the TCGA dataset. **(E, F)** The distribution of risk score and survival status for the ICGC dataset. **(G)** Survival analysis between the high and low risk groups for the ICGC dataset. **(H)** ROC curves showing the predictive value of the risk score for the ICGC dataset.

According to the median risk score, patients from the TCGA cohort were divided into the two groups. Figures 3A, B showed the distribution between risk scores and patients’ prognosis. By Kaplan-Meier analysis, the results showed that patients in the high-risk group had worse overall survival (OS) (Figure 3C). Further, the time-dependent ROC results indicated that the 1, 2, and 3-year AUC values were 0.745, 0.661, and 0.626, respectively (Figure 3D).

Furthermore, we assessed the predictive value of the gene signature in the ICGC HCC dataset. ICGC patients were also divided into different groups by applying the same median risk score from the TCGA dataset. Similar to the result of the TCGA dataset, the high risk group indicated poorer OS (Figures 3E–G), and the AUC value was 0.669 for 1 year, 0.677 for 2 years, and 0.650 for 3 years (Figure 3H).

### Independent prognostic value of the risk score

**FIGURE 4:**
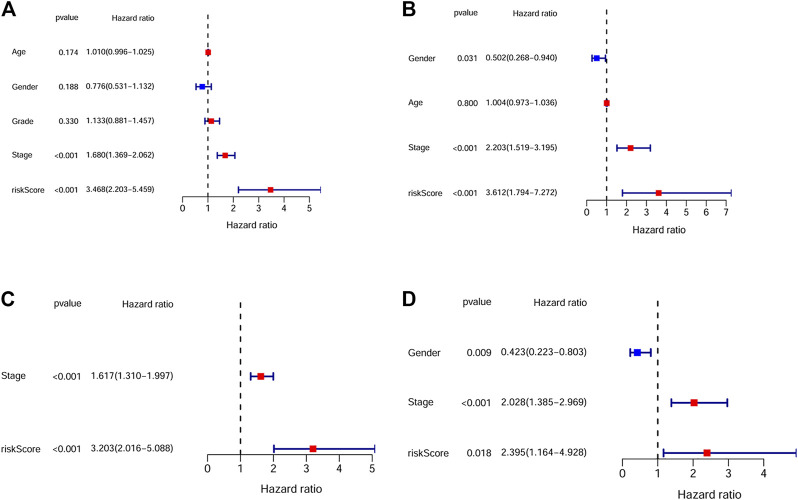
The independent prognostic value of the risk score. **(A)** Univariate cox regression analysis for the TCGA dataset. **(B)** Univariate cox regression analysis for the ICGC dataset. **(C)** Multivariate cox regression analysis for the TCGA dataset. **(D)** Multivariate cox regression analysis for the ICGC dataset.

**FIGURE 5:**
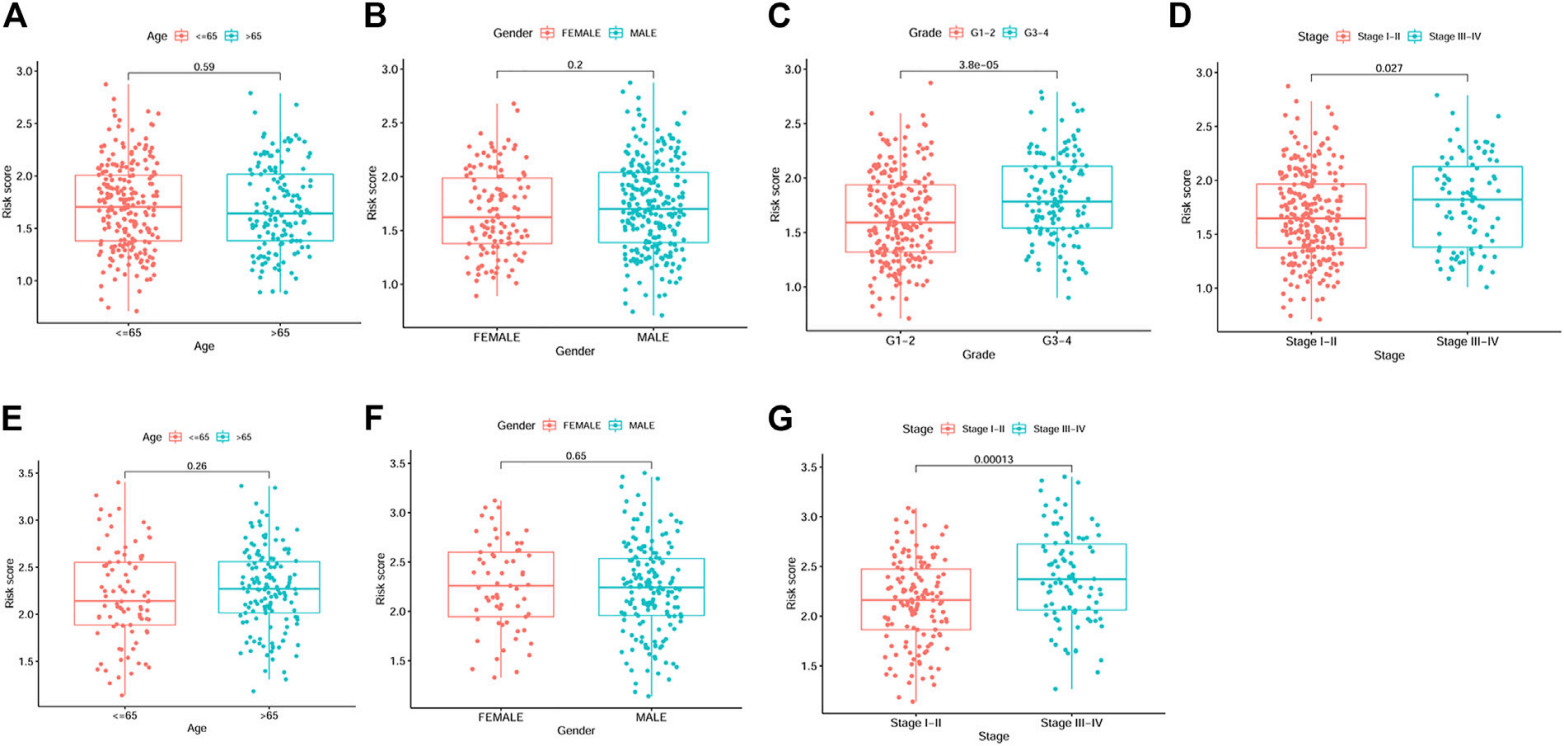
Box plots showing the correlations between risk score and clinicopathological factors. **(A–D)** Age, gender, grade and stage from TCGA dataset. **(E–G)** Age, gender and stage from ICGC dataset.

By univariate Cox analysis, our results showed that the risk score was significantly correlated with patients’ survival (For TCGA cohort: HR = 1.68, 95% CI = 1.369–2.062, *p* < 0.001; For ICGC cohort: HR = 2.203, 95% CI = 1.519–3.195, *p* < 0.001; Figures 4A, B). By multivariable Cox regression analysis, the risk score was proved to be an independent risk factor for both the training and validation cohort (For TCGA cohort: HR = 3.203, 95% CI = 2.016–5.088, *p* < 0.001; For ICGC cohort: HR = 2.395, 95% CI = 1.164–4.928, *p* = 0.018; Figures 4C, D). We also evaluated the associations between the risk score and common clinical parameters. Our results showed that the risk score was significantly associated with tumor grade and tumor stage (Figures 5C, D, G).

### Functional enrichment analysis between the low and high risk groups

**FIGURE 6:**
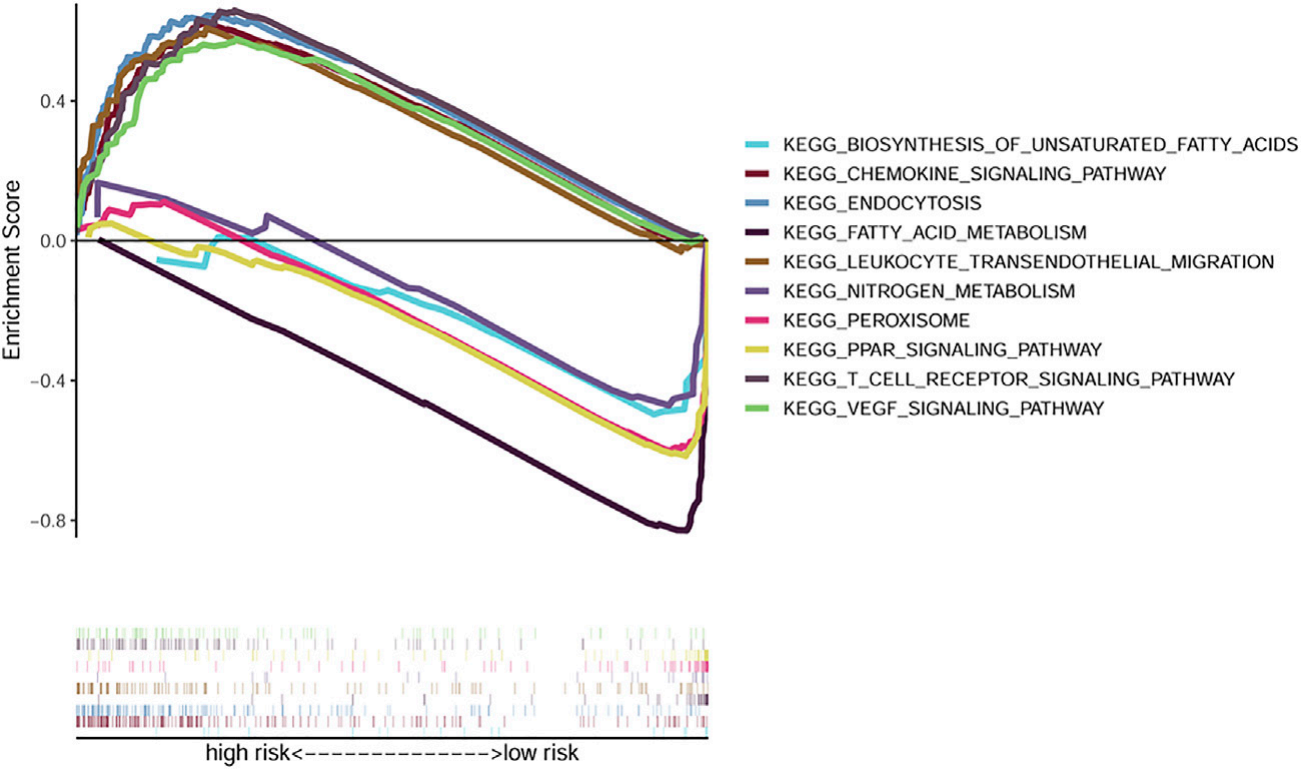
GSEA analysis exhibiting the top 5 enriched KEGG pathways in the high and low risk groups.

Results of the GSEA analysis showed the 50 most significantly enriched KEGG pathways. The endocytosis, chemokine signaling, leukocyte transendothelial migration, and VEGF signaling pathways were enriched in the high risk group. While peroxisome, peroxisome proliferator-activated receptors, fatty acid metabolism, and nitrogen metabolism pathways were enriched in the low risk group (Figure 6).

### Immune infiltration and TME analysis

**FIGURE 7:**
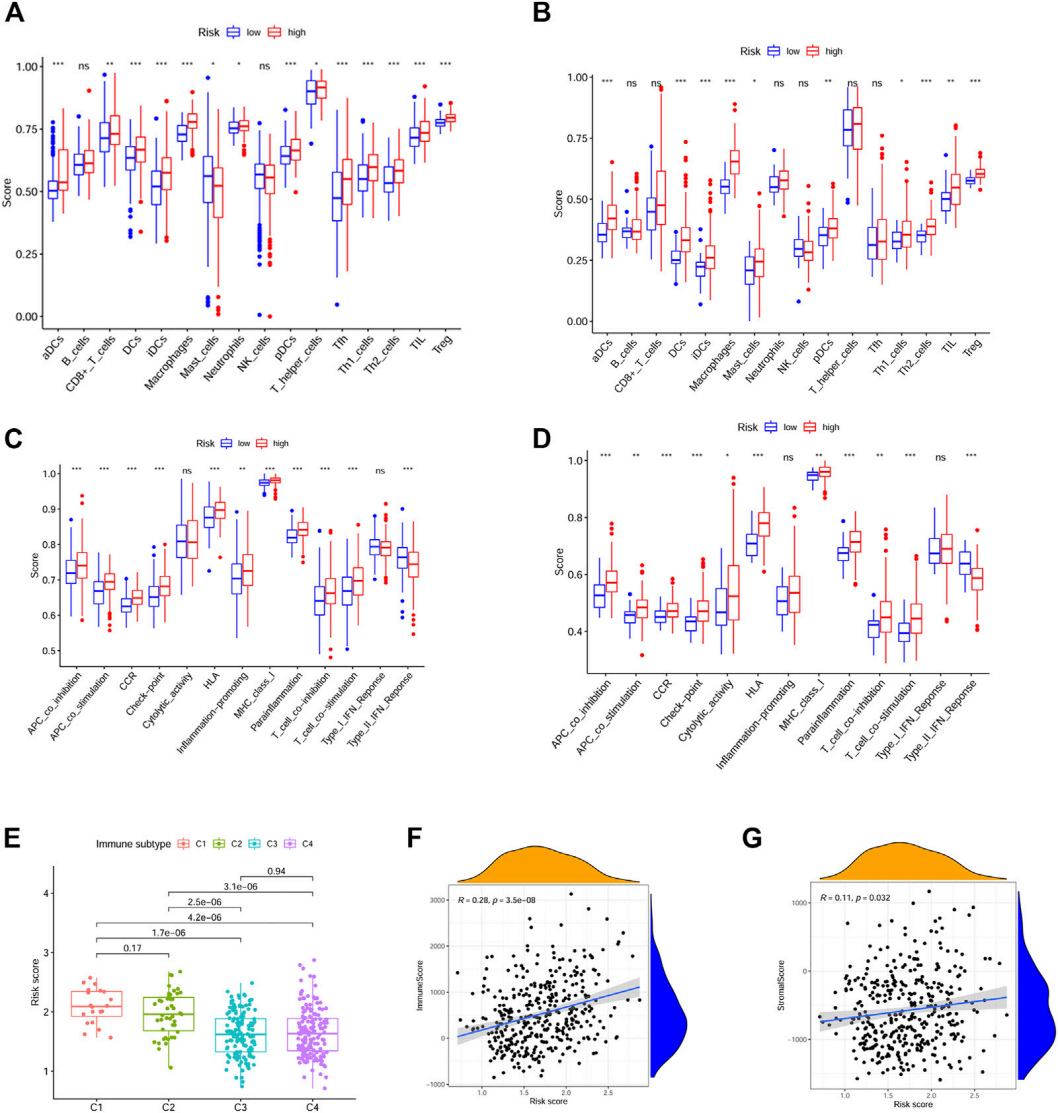
Immune infiltration between the high and low risk groups and drug resistance analysis. **(A, C)** Immune cells and immune-related pathways between the high-risk and low-risk groups in the TCGA dataset. **(B, D)** Immune cells and immune-related pathways between the high-risk and low-risk groups in the ICGC dataset. **(E)** Correlations between risk score and different immune subtypes. **(F, G)** Correlations between risk score and immune score, stromal score. (*p* values were represented as: ns not significant; *p < 0.05; **p < 0.01; ***p < 0.001.)

By ssGSEA, we further analyzed the correlations between risk score and immune infiltration. We calculated the scores of different immune cells and immune-related pathways. Our results indicated that higher levels of infiltration of aDCs, DCs, iDCs, pDCs, macrophages and Treg were observed in the high risk group both in the TCGA and ICGC cohorts, while the APC co-inhibition, T cell co-inhibition pathways were also activated in these groups. Interestingly, the type II IFN response was inhibited in the high risk group both in the TCGA and ICGC cohorts (Figures 7A–D).

**FIGURE 8:**
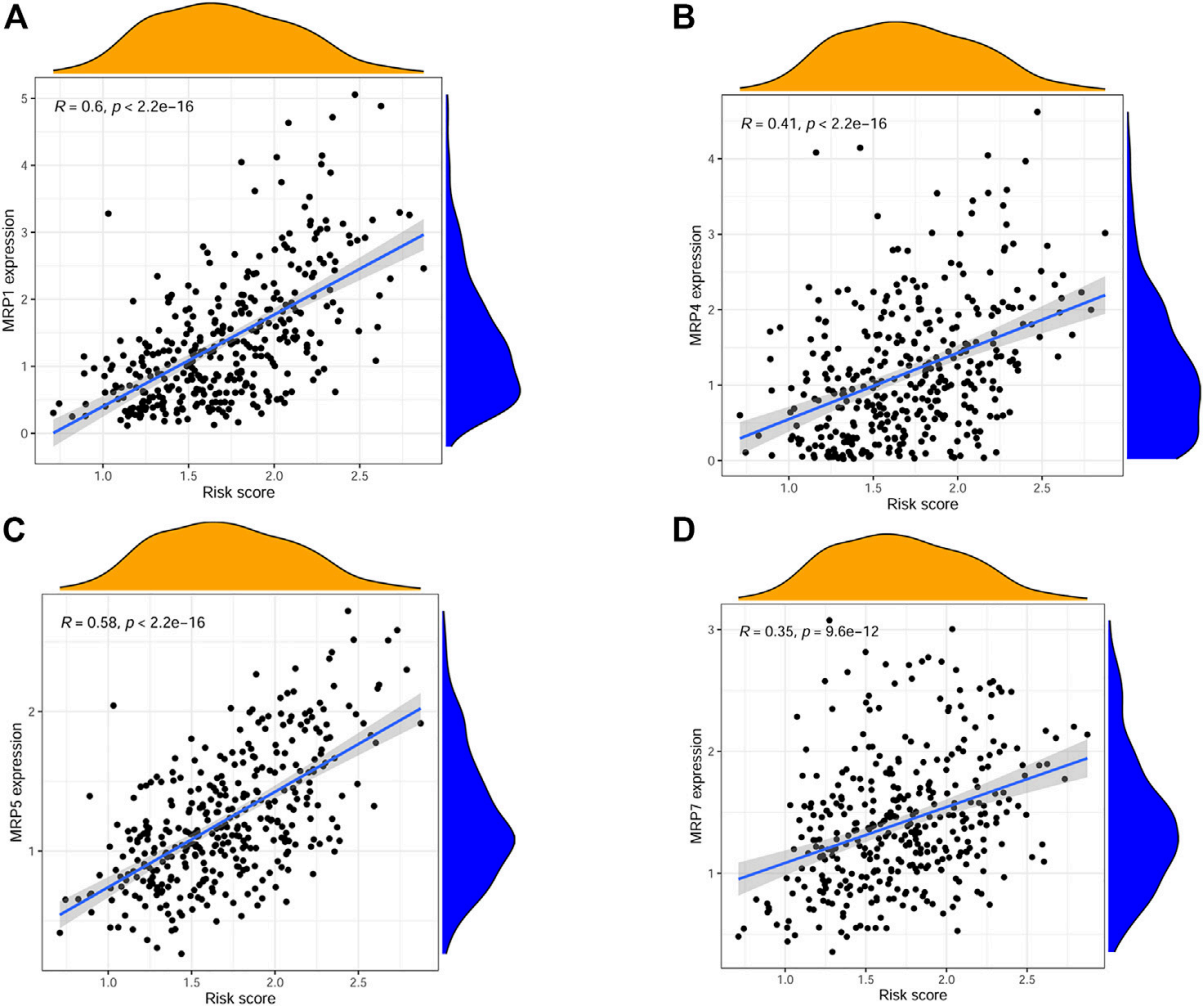
Scatter plots showing the relationship between risk score and MRP1, MRP4, MRP5, MRP7. **(A–D)**.

The correlations between risk score and pan-cancer immune infiltration subtypes was further evaluated. As reported by David Tamborero, immune infiltrates in solid tumors may be divided into six types (C1-C6 types), which indicated the role from tumor promotion to tumor inhibition (Tamborero et al., 2018). Our results showed that high risk score correlated with tumor promotion subtype, while low risk score implied tumor inhibition subtype (Figure 7E). By applying the ESTIMATE analysis, our results showed that the risk score was weak positive correlated with the immune score and stromal score (Figures 7F, G). In addition, multidrug resistance (MDR) is one of the main causes for treatment failure in HCC, and multidrug resistance proteins (MRPs) mediated multidrug resistance in various cancers including HCC (Ding et al., 2022). Here, we also analyzed the correlation between the risk score and MRPs (MRP1-MRP9). The results showed that the risk score was positively correlated with MRP1, MRP4, MRP5, and MRP7, which indicated that patients in the low risk group may be more likely to derive benefit from chemotherapy or targeted therapy (Figure 8).

## Discussion

ScRNA-seq technologies have deeply changed the paradigm to explore the TME. TAMCs, as the main immune cell types, are enriched and highly heterogeneous in tumor tissues. Here, we adopted the scRNA-seq method to explore the roles of HCC related TAMCs. We firstly defined 317 HCC-specific myeloid cell marker genes through scRNA-seq analysis of the GEO dataset. Then a novel prognostic gene signature was developed and validated by the TCGA and ICGC dataset. Similar to our results, another myeloid cell marker gene related gene signature was proved to be prognostic and predictive of immunotherapy response for patients with HNSCC (Liu et al., 2021). Further, the risk score was proved to be correlated with tumor grade and stage. Patients in the high-risk group had shorter OS. Besides, the risk score was associated with a tumor promotion environment. In addition, the risk score was also proved to be positively associated with drug resistance.

Our gene signature contained 8 myeloid cell related genes (FABP5, C15orf48, PABPC1, TUBA1B, AKR1C3, NQO1, AKR1B10, SPP1). All these genes were shown to be overexpressed in HCC tissues and also to be prognostic. 1) FABP5 takes part in fatty acids delivery as an intracellular carrier (Kaczocha et al., 2012). Accumulating evidence has suggested that FABP5 is commonly upregulated in most human malignancies. FABP5 was overexpressed in HCC and involved in the proliferation, migration, and invasion of HCC cells through a FABP5/CREB/miR-889-5p/KLF9 axis (Tang et al., 2022). More recently, FABP5 in monocytes/macrophages was shown to promote lipid accumulation and induction of the inhibitory tumor microenvironment of HCC(Liu J. et al., 2022). 2) C15orf48 is also known as NMES1, the function of this gene is poorly understood. Recently, C15orf48 was found to be overexpressed in response to activation of macrophages and involved in regulating inflammatory cytokines expression (Liu et al., 2009). C15orf48 was also proved to reduce tissue inflammation and immunity and proved to be protective during infection and inflammation (Lee et al., 2021). 3) PABPC1 was proved to take part in miRNA-mediated gene silencing including HCC(Tritschler et al., 2010; Zhang et al., 2015). Interestingly, PABPC1 was shown to regulate immunoglobulin secretion in immune cells (Peng et al., 2017). 4) TUBA1B, encodes the protein of tubulin alpha-1B chain, is the major constituent of microtubules. Study has shown that changes in the relative content of tubulin may regulate neutrophils activation (Rothwell et al., 1993). 5&6) AKR1C3 and AKR1B10 are enzymes that catalyzes redox transformation and play important role in tumor progression (Penning, 2015). AKR1C3 promoted HCC cells proliferation and metastasis through the AKR1C3/NF-κB/STAT3 axis, and was upregulated in HCC tissues. High expression of AKR1C3 correlated with poor survival (Zhou et al., 2021). AKR1C3 also inhibited the ubiquitination of PARP1 and thus resulting in HCC cell proliferation and resistance to Cisplatin (Pan et al., 2022). Similarly, AKR1B10 also promoted HCC progression and drug resistance (Zhang et al., 2022). 7) NQO1 is a flavoprotein, which is important in the cellular response to numerous stresses (Lee et al., 2015). NQO1 was shown to be overexpressed in HCC and correlated with poor survival (Lin et al., 2017). Mechanistically, NQO1 functioned as an agonist at pathways of PI3K/Akt and MAPK/ERK, which promoted HCC cell proliferation and tumor growth (Dimri et al., 2020). 8) SPP1 is a multifunctional gene, which takes part in a variety of cellular processes including cell epithelial transformation, a cytokine participating in the regulation of IL-12/IFN-γ, and the promotion of tumorigenesis (Ashkar et al., 2000; Han et al., 2019; Kashani-Sabet et al., 2017). Interestingly, SPP1 was also proved to be overexpressed in HCC and correlated with patients’ survival as well as an indicator for immunotherapy. More importantly, SPP1 may promote macrophages transition *via* SPP1-CD44 signaling (Liu L. et al., 2022;Liu Z. et al., 2022;Wang et al., 2019). In glioblastoma, SPP1 was found to be responsible for neutrophil and macrophage infiltration (Atai et al., 2011). Similarly, in this study, we found that SPP1 was positively correlated with myeloid cell infiltration but negatively correlated with CD4/CD8 cell infiltration. In summary, our findings of the signature genes may provide new therapeutic and prognostic targets for HCC.

The association between risk score and immune infiltrations were further explored. We firstly explored its role with the previously reported pan-cancer immune infiltration subtypes (Tamborero et al., 2018). Our results indicated that higher risk score positively correlated with C1 tumor promoting subtype. Next, we adopted the ssGSEA for evaluating the correlations between risk score and immune cell infiltration. Our study indicated that higher levels of DCs, Treg, and macrophages infiltrations were observed in the high-risk group. Besides, the APC co-inhibition, T cell co-inhibition pathways were also activated in the high-risk group. However, the type II IFN response was inhibited. It is widely recognized that a high abundance of Treg contributes to immunosuppression and tumor progression, as well as the tumor associated macrophages (Deng et al., 2021; Zhang et al., 2019). Consistently, type II IFN response has played important roles in tumor surveillance, and IFN-γ, as the only executor of type II IFN response and the most important macrophage stimuli, can induce direct antimicrobial and antitumor effects (Petermann et al., 2019; Schroder et al., 2004). Moreover, through ESTIMATE analysis, our results showed that the risk score did not significantly correlated with both the immune score and stromal score, which needed to be verified in future studies. We next analyzed the pathways changed between the different groups by GSEA analysis. And the data showed that the endocytosis, chemokine signaling, leukocyte transendothelial migration and VEGF signaling pathways activated in the high-risk group. While fatty acid metabolism, peroxisome, peroxisome proliferator-activated receptors and nitrogen metabolism pathways were enriched in the other group.

Chemical or targeted drugs are important method for HCC treatment. For example, sorafenib and lenvatinib were all recommended as first-line therapies for unresectable hepatocellular carcinoma (Cheng et al., 2009; Kudo et al., 2018). However, MDR seriously affected the treatment efficacy. The MRPs are the well-known transporters, which takes part in multidrug resistance through extruding drugs from cancer cells (Sodani et al., 2012). Here, our results showed that the risk score was positively correlated with MRP1, MRP4, MRP5, and MRP7. Our data indicated that the low-risk group may be more sensitive to chemical or targeted therapy.

Nevertheless, our study has some limitations. Firstly, we only validated the expression of SPP1 in HCC tissues. As all the analyses retrieved is from public sources, further experimental studies are still needed to verify these findings. Secondly, due to the limitation of scRNA-seq technique, a depth coverage of rare cell types was restricted. We could not clearly distinguish neutrophils/macrophage subtypes with known marker genes. Besides, all the included genes were derived from myeloid cell marker genes, while the TME was rather heterogeneous. So, the prognostic predicting value may be inhibited. Last but not least, the underlying connections for these genes between TME and tumor cells deserved to be investigated further.

In conclusion, we constructed a gene signature from HCC specific myeloid cell marker genes and this gene signature was proved to be valuable in immune infiltration analysis, drug resistance prediction and prognostic prediction. The study exhibits valuable knowledge regarding the roles of myeloid cell marker genes in HCC. Our findings may be useful for individualized treatment decisions making.

## Data Availability

The original contributions presented in the study are included in the article/Supplementary Material, further inquiries can be directed to the corresponding authors.
