# Supplementary figures and images for: A novel myeloid cell marker genes related signature can indicate immune infiltration and predict prognosis of hepatocellular carcinoma: Integrated analysis of bulk and single-cell RNA sequencing
Source: Front Mol Biosci. 2023 Mar 7;10:1118377. doi: 10.3389/fmolb.2023.1118377 (PMC10027926; doi:10.3389/fmolb.2023.1118377)

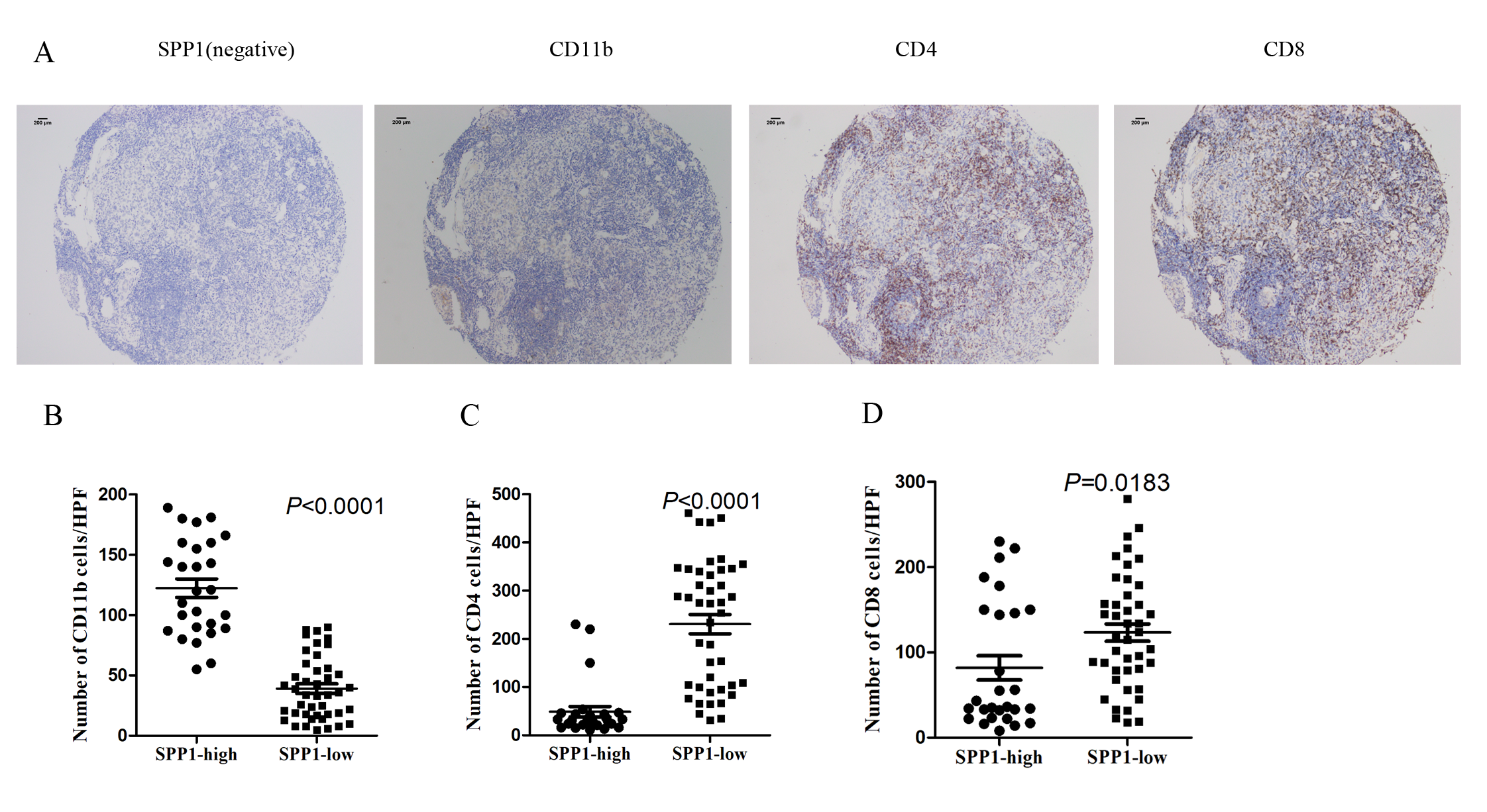

Supplement: Supplementary file 1 [file Image3.TIF]

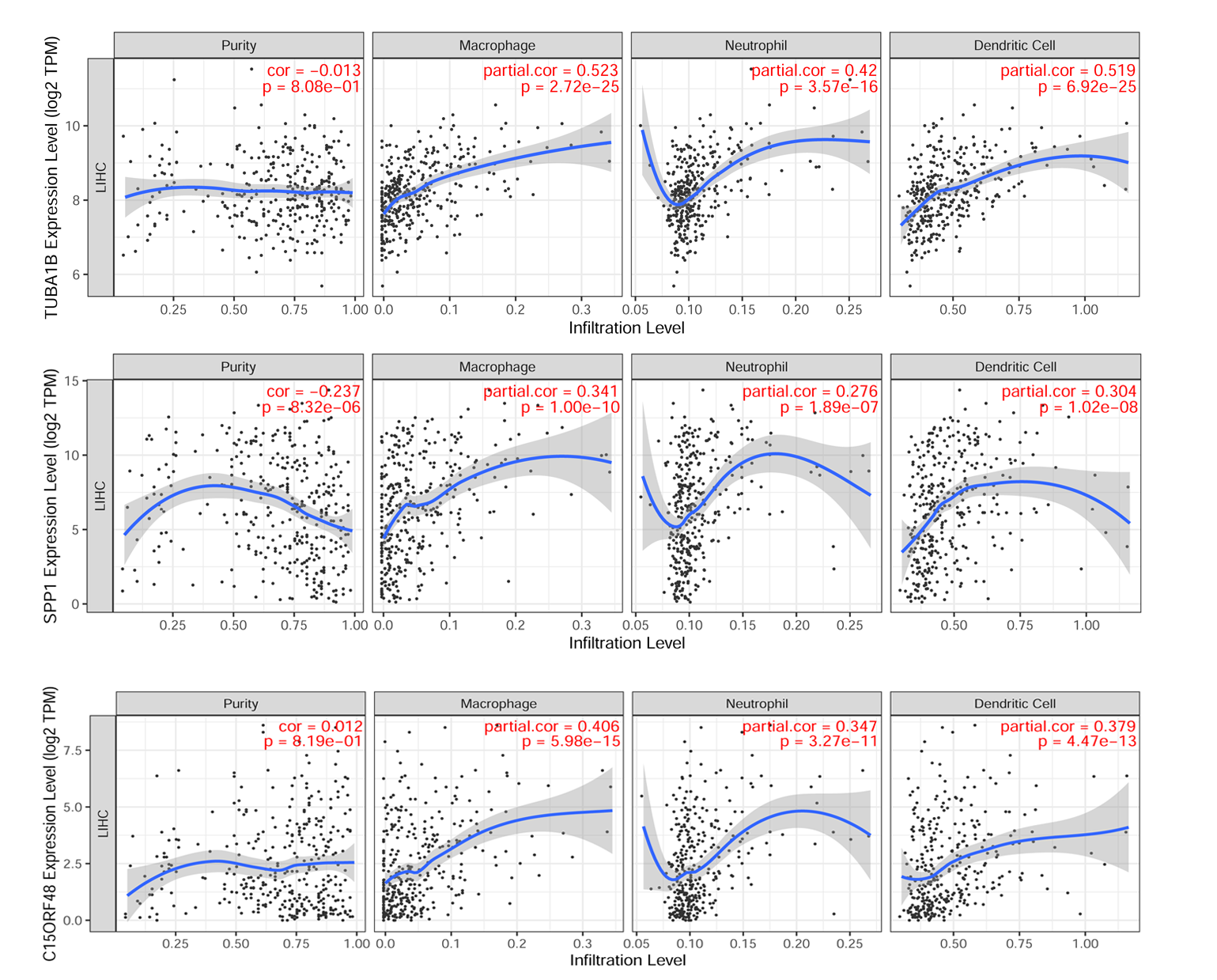

Supplement: Supplementary file 2 [file Image2.TIF]

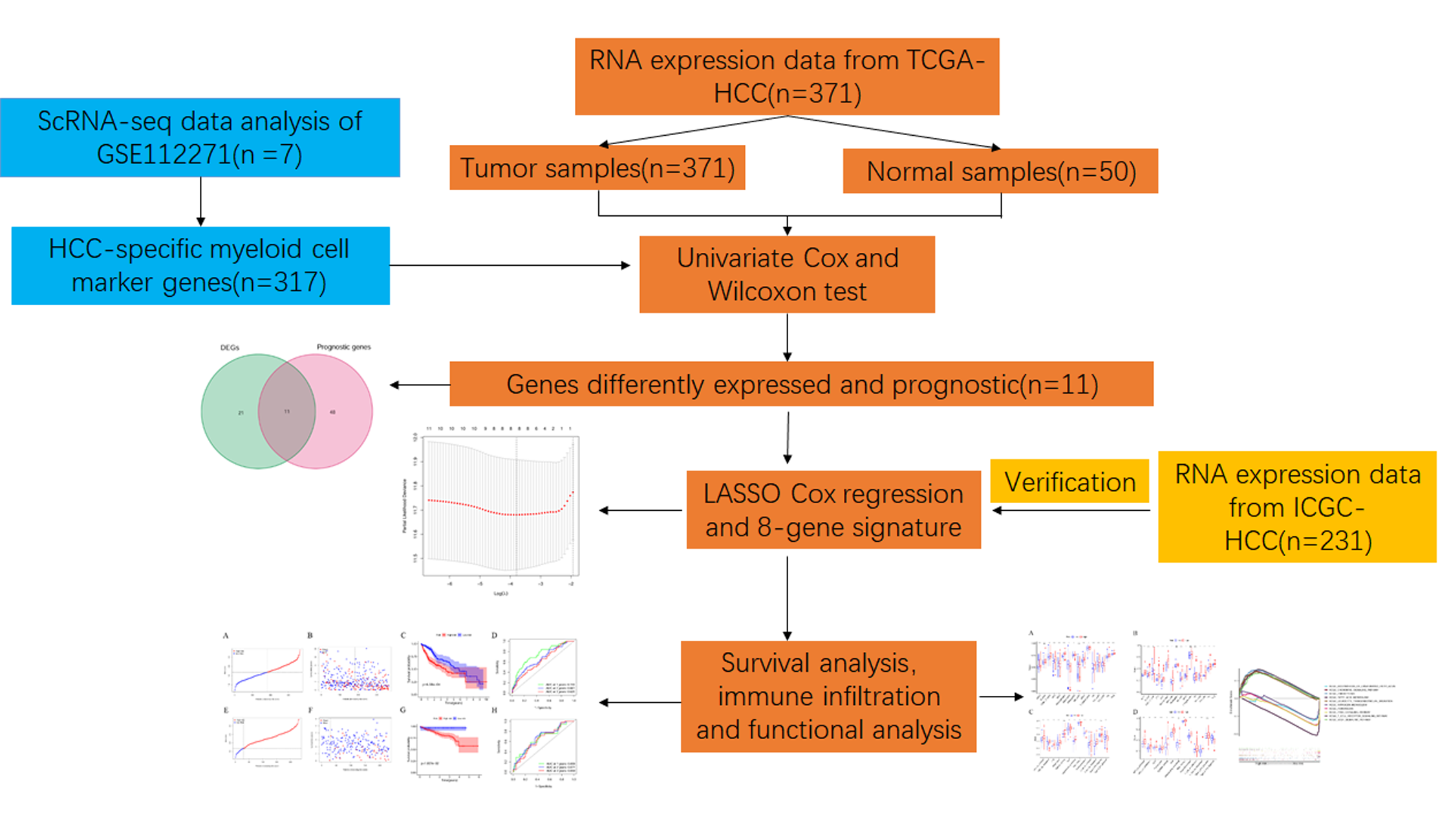

Supplement: Supplementary file 3 [file Image1.TIF]
